# NDM-Producing Klebsiella pneumoniae and Multidrug-Resistant Enterococcus faecium Bacteremia: A Report of Two Cases

**DOI:** 10.7759/cureus.109960

**Published:** 2026-05-31

**Authors:** Nikolaos Iason Davanellos, Polyxeni Stefa, Rafail Giannas, Dimitrios Peichamperis, Despoina Paraskeva

**Affiliations:** 1 Department of Internal Medicine, General Hospital of Syros, Ermoupoli, GRC; 2 Department of Microbiology, General Hospital of Syros, Ermoupoli, GRC; 3 Department of Radiology, General Hospital of Syros, Ermoupoli, GRC

**Keywords:** antimicrobial resistance, bloodstream infection, ceftazidime–avibactam, enterococcus faecium, extensively drug-resistant klebsiella pneumoniae, linezolid resistance, ndm carbapenemase, urosepsis

## Abstract

Antimicrobial resistance (AMR) has emerged as a major global healthcare challenge, significantly complicating the management of healthcare-associated infections and limiting available therapeutic options. We present two cases of severe bloodstream infections caused by multidrug-resistant pathogens in patients with prolonged healthcare exposure and multiple comorbidities. The first case involved urinary-source bacteremia caused by NDM (New Delhi metallo-β-lactamase)-producing *Klebsiella pneumoniae* exhibiting extensive AMR. Despite limited therapeutic options and apparent *in vitro* resistance, treatment with ceftazidime-avibactam was associated with marked clinical and laboratory improvement, resulting in clinical recovery and hospital discharge. The second case involved urinary-source bacteremia caused by multidrug-resistant *Enterococcus faecium* in an elderly patient with significant comorbidities and recent broad-spectrum antimicrobial exposure. Despite escalation of antimicrobial therapy and supportive management, the patient experienced progressive clinical deterioration and ultimately died. These cases highlight the substantial therapeutic challenges posed by highly resistant pathogens and emphasize the importance of early microbiological diagnosis, individualized interpretation of antimicrobial susceptibility results, prompt optimization of antimicrobial therapy, and strict infection prevention measures in the management of severe multidrug-resistant infections.

## Introduction

Antimicrobial resistance (AMR) has emerged as one of the most significant global public health threats, contributing substantially to increased morbidity, mortality, and healthcare costs worldwide [1]. The rapid global dissemination of multidrug-resistant (MDR) organisms has significantly complicated the management of hospital-acquired infections, particularly among critically ill or immunocompromised patients [2]. Recent epidemiological data have demonstrated a marked global increase in carbapenem-resistant *Enterobacterales* (CRE) and vancomycin-resistant enterococci (VRE), particularly in healthcare-associated infections among critically ill and heavily healthcare-exposed patients [3].

Among Gram-negative pathogens, *Klebsiella pneumoniae* has become one of the most clinically important causes of healthcare-associated infections. In particular, carbapenem-resistant *Klebsiella pneumoniae* (CRKP) represents a major therapeutic challenge due to resistance to multiple antimicrobial classes and its association with high mortality rates, especially in cases of bloodstream infection [4]. The introduction of novel β-lactam/β-lactamase inhibitor combinations, including ceftazidime-avibactam, has expanded available therapeutic strategies for selected CRE infections and has demonstrated favorable clinical outcomes in several studies [5].

Meanwhile, Gram-positive pathogens remain a significant contributor to AMR in healthcare settings. *Enterococcus faecium*, particularly vancomycin-resistant strains (VRE), has emerged as a major cause of nosocomial infections, including bloodstream infections, urinary tract infections, and intra-abdominal infections [6]. Linezolid has traditionally been considered one of the most important therapeutic options for VRE infections; however, the emergence of linezolid-resistant enterococci represents an additional clinical challenge due to the limited availability of effective alternative therapies [7,8].

In this report, we present two cases of bloodstream infection caused by MDR pathogens managed in a secondary care hospital, illustrating the therapeutic challenges, microbiological complexity, and contrasting clinical outcomes associated with MDR Gram-negative and Gram-positive infections.

## Case presentation

Two cases of severe infections caused by MDR pathogens were identified in patients hospitalized in a secondary care hospital.

Case 1

A 53-year-old female was admitted to the internal medicine department of a secondary care hospital due to reported fever. Her medical history was significant for arterial hypertension, type 2 diabetes mellitus, ischemic heart disease, chronic heart failure, and multiple recent cerebrovascular events resulting in severe neurological impairment and prolonged healthcare exposure. The patient had recurrent hospitalizations, a tracheostomy, enteral feeding through a nasogastric tube, chronic pressure ulcers, and an indwelling urinary catheter at the time of admission, all of which increased the risk for healthcare-associated MDR infections.

Upon admission, the patient had a temperature of 37.2°C, blood pressure of 139/95 mmHg, and heart rate of 93 beats per minute. Oxygen saturation was 85% on room air. Oxygen therapy with a Venturi mask delivering 50% fraction of inspired oxygen (FiO₂) increased oxygen saturation to 99%, after which respiratory support was de-escalated to a nasal cannula at 5 L/min, maintaining adequate oxygenation.

Initial laboratory evaluation demonstrated markedly elevated inflammatory markers, severe hyperglycemia, anemia, elevated liver and cholestatic enzymes, and mildly elevated high-sensitivity troponin without ischemic electrocardiographic changes (Table 1).

**Table 1:** Evolution of laboratory parameters during hospitalization - case 1. Laboratory parameters of case 1 at admission, during hospitalization, and at discharge, demonstrating changes in inflammatory markers, glycemic status, hematologic indices, and liver function following antimicrobial therapy.

Parameter	Reference range	Admission	During hospitalization	Discharge
White blood cells (×10⁹/L)	4-10	9.55	7.20	7.36
Neutrophils (%)	40-75	88.3	75	71
C-reactive protein (mg/L)	<5	318	80	20.8
Procalcitonin (ng/mL)	<0.05	5.6	1.15	0.15
Hemoglobin (g/dL)	12-16	10.6	7.8	9.2
Hematocrit (%)	36-46	34	25.8	30.1
Blood glucose (mg/dL)	70-140	416	220	140
Aspartate aminotransferase (U/L)	<35	534	22	12
Alanine aminotransferase (U/L)	<45	931	96	16
Alkaline phosphatase (U/L)	40-130	365	202	181
γ-glutamyl transferase (U/L)	<55	264	285	191
Lactate dehydrogenase (U/L)	135-225	465	278	148
High-sensitivity troponin (pg/mL)	<14	116	102	26

A computed tomography (CT) scan of the brain, chest, and abdomen was performed upon admission. Brain imaging demonstrated chronic ischemic and gliotic changes consistent with the patient’s known cerebrovascular disease. Abdominal imaging revealed gallbladder wall thickening exceeding 4 mm and a distended gallbladder containing intraluminal hyperdense content compatible with cholelithiasis (Figure 1). In addition, peripancreatic fat stranding and a small fluid collection in the hepatorenal recess (Morison’s pouch) were observed, supporting the presence of an intra-abdominal inflammatory process.

**Figure 1:**
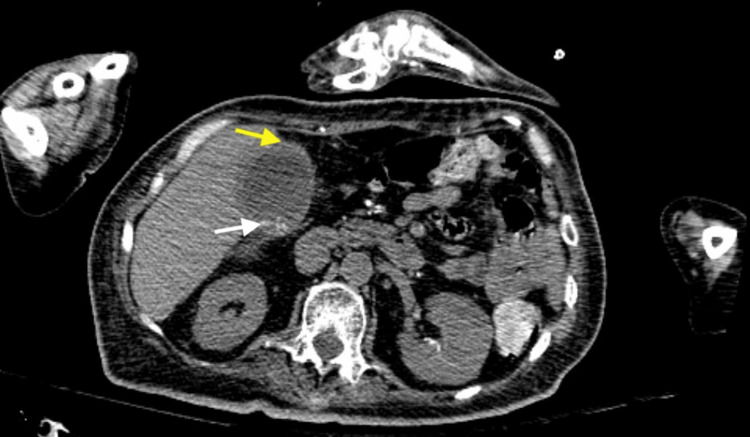
Abdominal CT findings in case 1. Axial abdominal CT (soft tissue window) demonstrating gallbladder wall thickening (yellow arrow) and intraluminal hyperdense content within a distended gallbladder (white arrow), with imaging findings suggestive of acute cholecystitis.

Given the markedly elevated inflammatory markers and the suspicion of systemic infection, blood and urine cultures were obtained prior to the initiation of antimicrobial therapy. Empirical antimicrobial therapy with intravenous piperacillin-tazobactam was initiated upon admission at full therapeutic dosing.

Microbiological evaluation subsequently revealed growth of *Klebsiella pneumoniae* in both blood and urine cultures, consistent with bacteremia. Antimicrobial susceptibility testing demonstrated an MDR phenotype with resistance to multiple antimicrobial classes. The isolate was identified as an NDM (New Delhi metallo-β-lactamase) carbapenemase-producing *Klebsiella pneumoniae*, exhibiting resistance to multiple antimicrobial classes, including carbapenems, fluoroquinolones, aminoglycosides, colistin, and piperacillin-tazobactam (Table 2). Notably, the organism also demonstrated *in vitro* resistance to ceftazidime-avibactam.

**Table 2:** Antimicrobial susceptibility profile of the isolates. R: resistant; N/A: not available. Antimicrobial susceptibility testing and interpretation were performed using automated microbiological methods according to the European Committee on Antimicrobial Susceptibility Testing (EUCAST) breakpoints [9] and institutional microbiology laboratory protocols.

Antibiotic	*Klebsiella pneumoniae* (Case 1)	*Enterococcus faecium* (Case 2)
Ampicillin	R	R
Piperacillin-tazobactam	R	–
Ciprofloxacin	R	R
Levofloxacin	R	–
Gentamicin	R	R
Amikacin	R	–
Imipenem	R	–
Meropenem	R	–
Ertapenem	R	–
Colistin	R	–
Vancomycin	–	R
Teicoplanin	–	R
Linezolid	–	R
Tigecycline	N/A	R
Trimethoprim-sulfamethoxazole	R	–
Ceftazidime-avibactam	R	–

Given the limited available therapeutic options and the patient’s severe clinical condition with prolonged healthcare exposure, antimicrobial therapy was modified on the third day of hospitalization to ceftazidime-avibactam combined with linezolid as salvage therapy, aiming to provide broad coverage for possible concomitant MDR Gram-positive pathogens while awaiting complete microbiological evaluation. Ceftazidime-avibactam treatment was continued for a total duration of eight days.

Following modification of antimicrobial therapy to a salvage regimen, the patient demonstrated rapid clinical improvement. Fever resolved shortly after modification of antimicrobial therapy. Inflammatory markers progressively improved during hospitalization, while leukocyte counts remained within normal limits throughout the hospital stay (Table 1).

During hospitalization, worsening anemia developed, requiring transfusion of one unit of packed red blood cells, after which hemoglobin levels stabilized (Table 1).

The total duration of hospitalization was 15 days, after which the patient was discharged in stable clinical condition.

Case 2

A 100-year-old male with multiple comorbidities was admitted to the internal medicine department of a secondary care hospital due to fever and progressive respiratory deterioration. The patient had been hospitalized 10 days prior for multiple rib fractures complicated by hemothorax, as well as lower respiratory tract infection, during which he received broad-spectrum antimicrobial therapy with piperacillin-tazobactam. Recent hospitalization and broad-spectrum antimicrobial exposure represented significant risk factors for healthcare-associated MDR infection. His past medical history was notable for Parkinson’s disease.

Upon admission, the patient exhibited high fever (39.6°C) and severe hypoxemia. Oxygen therapy with a non-rebreather mask delivering 100% oxygen was required to maintain oxygen saturation around 92%. Vital signs revealed blood pressure of 121/84 mmHg and heart rate of 107 beats per minute. Blood and urine cultures were obtained on admission prior to the initiation of antimicrobial therapy.

Chest computed tomography (CT) performed upon admission demonstrated a large left-sided pleural fluid collection compatible with residual hemothorax and multiple rib fractures related to the recent traumatic injury (Figure 2). These findings were interpreted in the context of the patient’s recent hospitalization and underlying respiratory compromise.

**Figure 2:**
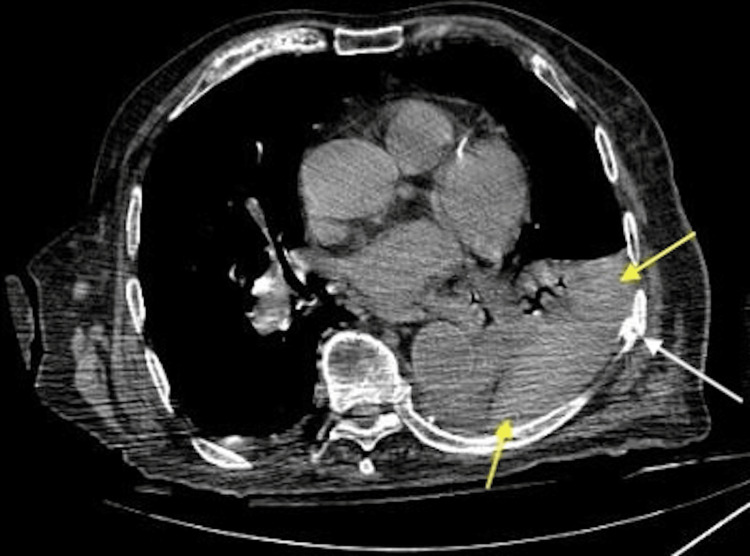
Chest CT findings in case 2. Axial chest CT in soft tissue window demonstrating a large left-sided pleural fluid collection compatible with residual hemothorax (yellow arrows) and associated rib fractures (white arrow).

Initial laboratory evaluation demonstrated marked inflammatory response with leukocytosis and neutrophilia, hypoalbuminemia, elevated D-dimer levels, and abnormal urinalysis findings, compatible with severe urinary tract infection (Table 3).

**Table 3:** Evolution of laboratory parameters during hospitalization - case 2. Laboratory investigations of case 2 demonstrated persistent inflammatory activity and progressive clinical deterioration despite antimicrobial therapy.

Parameter	Reference range	Admission	During hospitalization	Final value
White blood cells (×10⁹/L)	4-10	15.07	12.97	17.65
Neutrophils (%)	40-75	83.1	90.3	89.6
C-reactive protein (mg/L)	<5	138	281	330
Procalcitonin (ng/mL)	<0.05	—	0.8	1.61
Urea (mg/dL)	15-50	71	54	50
Creatinine (mg/dL)	0.7-1.3	0.88	0.63	0.68
Albumin (g/dL)	3.5-5.0	2.9	2.8	2.9
Lactate dehydrogenase (U/L)	135-225	348	247	263
D-dimers (ng/mL)	<500	2519	—	—
Urine leukocytes (/high-power field)	0-5	30-40	Persistent pyuria	—
Urine erythrocytes (/high-power field)	0-2	35-45	Persistent hematuria	—

Urinalysis demonstrated persistent pyuria (30-40 leukocytes/high-power field (HPF)) and microscopic hematuria (35-45 erythrocytes/HPF) (Table 3). Although pyuria may be nonspecific in very elderly patients, particularly in the presence of urinary catheterization, the concurrent isolation of *Enterococcus faecium* from both urine and blood cultures supported a urinary source of bacteremia in the present case. The patient had an indwelling urinary catheter, and urine cultures also yielded *Candida spp*. Given the absence of candidemia, obstructive fungal lesions, or imaging findings suggestive of invasive fungal urinary tract infection, candiduria was interpreted cautiously and was considered more likely to reflect colonization associated with urinary catheterization, advanced age, and prolonged healthcare exposure rather than definite invasive fungal infection.

Empirical antimicrobial therapy with intravenous piperacillin-tazobactam was initiated on the first day of hospitalization. Due to clinical deterioration and microbiological findings, antimicrobial therapy was subsequently escalated to meropenem, linezolid, and fluconazole. Given the patient’s severe clinical deterioration, persistent fever, polymicrobial urinary findings, and concern for possible complicated urinary tract infection in the setting of prolonged urinary catheterization, broad-spectrum antimicrobial escalation with meropenem and adjunctive fluconazole was undertaken despite the uncertain pathogenic significance of candiduria.

Antimicrobial susceptibility testing of the isolates from both blood and urine cultures revealed an MDR *Enterococcus faecium* phenotype, demonstrating resistance to multiple antimicrobial classes, including ampicillin, ciprofloxacin, vancomycin, teicoplanin, linezolid, and tigecycline (Table 2).

The total duration of hospitalization was six days, during which the patient demonstrated progressive clinical deterioration. Persistent elevation of inflammatory markers and leukocytosis with neutrophilia remained evident throughout hospitalization despite antimicrobial therapy (Table 3). Procalcitonin levels also remained elevated, supporting ongoing severe systemic infection.

Repeat blood and urine cultures obtained during hospitalization were negative, suggesting microbiological clearance. Nevertheless, the patient remained persistently febrile, with daily fever episodes and a maximum recorded temperature of 39.6°C. Respiratory support remained necessary, and oxygen therapy was continued with a Venturi mask and nasal cannula following initial stabilization.

Despite aggressive antimicrobial therapy and supportive management, the patient’s clinical condition progressively deteriorated, ultimately leading to death on the sixth day of hospitalization.

The main clinical characteristics, antimicrobial resistance profiles, therapeutic approaches, and outcomes of the two cases are summarized in Table 4.

**Table 4:** Clinical characteristics of the two cases.

Variable	Case 1	Case 2
Age	53 years	100 years
Sex	Female	Male
Comorbidities	Diabetes mellitus, hypertension, ischemic heart disease, heart failure, multiple strokes	Parkinson’s disease, recent rib fractures with hemothorax
Healthcare exposure	Recent ICU hospitalization and rehabilitation facility stay	Recent hospitalization
Source of infection	Urinary tract infection with bacteremia	Urinary tract infection with bacteremia
Pathogen	Klebsiella pneumoniae	Enterococcus faecium
Resistance phenotype	Pandrug-resistant (PDR), NDM-producing isolate	Multidrug-resistant
Initial therapy	Piperacillin-tazobactam	Piperacillin-tazobactam
Targeted therapy	Ceftazidime-avibactam + linezolid	Meropenem + linezolid + fluconazole
Clinical outcome	Clinical improvement and discharge	Death

## Discussion

The global increase in infections caused by MDR and extensively drug-resistant (XDR) organisms represents a major challenge for modern clinical practice, particularly among patients with prolonged healthcare exposure and multiple comorbidities [10]. These infections are associated with increased mortality, prolonged hospitalization, and significant therapeutic limitations.

The first case describes bacteremia caused by an NDM-producing *Klebsiella pneumoniae* isolate exhibiting an XDR phenotype. CRKP has emerged as one of the most problematic pathogens in healthcare settings worldwide [11]. Among the various carbapenemases, the NDM is of particular concern due to its ability to hydrolyze most β-lactam antibiotics, including carbapenems, while remaining poorly inhibited by currently available β-lactamase inhibitors such as avibactam [12].

Infections caused by NDM-producing organisms are associated with extremely limited therapeutic options and high mortality rates [13]. Current treatment strategies are often based on combination regimens or salvage therapies, frequently guided by limited clinical evidence. Although current guidelines frequently recommend combination therapy with ceftazidime-avibactam plus aztreonam for infections caused by NDM-producing *Enterobacterales*, synergy testing with aztreonam was not available in the present case, reflecting local diagnostic and therapeutic limitations frequently encountered in secondary care settings [14]. In this context, the management of infections caused by NDM-producing *Klebsiella pneumoniae* remains particularly challenging.

Notably, in the present case, the patient demonstrated significant clinical improvement following the administration of ceftazidime-avibactam despite apparent *in vitro* resistance. Discordance between antimicrobial susceptibility testing and clinical response has been previously reported in the literature [15]. Several factors may contribute to this phenomenon, including pharmacodynamic effects, bacterial population heterogeneity, limitations of conventional susceptibility testing methods, and host-related factors, including immune response [16]. The decision to administer ceftazidime-avibactam despite documented in vitro resistance was based on the extremely limited therapeutic alternatives, the severity of infection, and the possibility that conventional susceptibility testing may not fully predict in vivo clinical response in selected cases. This observation is consistent with the well-described “90-60 rule” in clinical microbiology, whereby a proportion of infections caused by isolates reported as resistant may still demonstrate clinical response under specific clinical circumstances [17]. Similar discrepancies between microbiological susceptibility results and clinical outcomes have been previously described, supporting the importance of individualized clinical judgment in the management of severe MDR infections. However, interpretation of this observation should be cautious, as no additional molecular resistance testing or pharmacokinetic analyses were available to further explain the observed clinical response.

The second case illustrates the clinical difficulties associated with infections caused by MDR *Enterococcus faecium*. Enterococci are well-recognized nosocomial pathogens, particularly in patients with prolonged hospitalization, multiple comorbidities, and exposure to broad-spectrum antibiotics [5]. *Enterococcus faecium*, in particular, has demonstrated a remarkable capacity to acquire resistance to multiple antimicrobial agents, significantly limiting available treatment options.

Enterococcal bacteremia is associated with substantial morbidity and mortality, especially among elderly or critically ill patients [18]. Although candiduria in elderly catheterized patients frequently represents colonization rather than true invasive fungal infection, antifungal treatment may occasionally be considered in selected critically ill patients with persistent fever, significant clinical deterioration, extensive healthcare exposure, and polymicrobial urinary findings when invasive infection cannot be confidently excluded. In the specific case, despite the prompt initiation of broad-spectrum antimicrobial therapy and subsequent escalation of treatment, the patient experienced progressive clinical deterioration and ultimately died. This unfavorable outcome highlights the poor prognosis frequently associated with severe infections caused by MDR enterococci in vulnerable patient populations.

Taken together, the two cases presented here illustrate the complexity of managing severe infections caused by highly resistant pathogens. While the first case demonstrates that salvage antimicrobial therapy may occasionally result in favorable outcomes even in the presence of extensive antimicrobial resistance, the second case highlights the substantial mortality risk associated with severe MDR infections in frail patients.

These cases emphasize the importance of early microbiological diagnosis, individualized interpretation of antimicrobial susceptibility results, prompt optimization of antimicrobial therapy, and strict infection control measures in the management of severe MDR infections [19]. At the same time, the increasing prevalence of highly resistant pathogens underscores the urgent need for the development of novel antimicrobial agents and optimized therapeutic strategies.

## Conclusions

MDR infections caused by *Klebsiella pneumoniae* and *Enterococcus faecium* remain associated with substantial therapeutic challenges and high mortality, particularly in patients with prolonged healthcare exposure and multiple comorbidities. These two cases highlight the importance of early microbiological diagnosis, careful interpretation of antimicrobial susceptibility results, prompt optimization of antimicrobial therapy, and strict infection control measures. In addition, the observed clinical response despite apparent *in vitro* resistance in the first case underscores the complexity of managing severe MDR infections and the need for further research regarding optimal therapeutic strategies.
